# Fourth Controlled Human Infection Model (CHIM) meeting − CHIMs in endemic countries, May 22–23, 2023^☆^

**DOI:** 10.1016/j.biologicals.2024.101747

**Published:** 2024-02-13

**Authors:** Melissa Kapulu, Lucinda Manda-Taylor, Shobana Balasingam, Gary Means, Mikal Ayiro Malungu, Philip Bejon, Primus Che Chi, Christopher Chiu, E. Chandler Church, Rodrigo Correa-Oliveira, Nicholas Day, Anna Durbin, Moses Egesa, Claudia Emerson, Kondwani Jambo, Roli Mathur, Wolfram Metzger, Noni Mumba, Winfred Nazziwa, Ally Olotu, Jacqueline Rodgers, Frank Sinyiza, Kawsar Talaat, Ingrid Kamerling, Charlie Weller, Marc Baay, Pieter Neels

**Affiliations:** a KEMRI-Wellcome Trust Research Programme, Kenya; b Kamuzu University of Health Sciences, Malawi; c Wellcome Trust, London, UK; d Bill & Melinda Gates Foundation, Seattle, USA; e Pharmacy and Poisons Board, Kenya; f Imperial College London, UK; g Fred Hutchinson Cancer Center, Seattle, USA; h Fundação Oswaldo Cruz (Fiocruz), Brazil^2^; i Mahidol Oxford Tropical Medicine Research Unit, Bangkok, Thailand; j Johns Hopkins Bloomberg School of Public Health, Baltimore, USA; k MRC/UVRI and LSHTM Uganda Research Unit, Uganda; l McMaster University, Institute on Ethics & Policy for Innovation, Canada; m Malawi Liverpool Wellcome Programme, Malawi; n Bioethics Unit, Indian Council of Medical Research, India; o Eberhard Karls University, Tuebingen, Germany; p Uganda National Council for Science and Technology, Uganda; q Ifakara Health Institute, Tanzania; r AVAREF secretariat, WHO AFRO, Brazzaville, Congo; s National Health Sciences Research Committee, Malawi; t Centre for Human Drug Research, Leiden, the Netherlands; u P95 Epidemiology & Pharmacovigilance, Leuven, Belgium; v IABS-EU, Lyon, France; w London School of Hygiene and Tropical Medicine, UK

**Keywords:** Low- and middle-income countries, Infectious diseases, Human challenge, Deliberate infection, Vaccine, Human infection studies, Endemic diseases

## Abstract

Earlier meetings laid the foundations for Controlled Human Infection Models (CHIMs), also known as human challenge studies and human infection studies, including Good Manufacturing Practice (GMP) production of the challenge agent, CHIM ethics, environmental safety in CHIM, recruitment, community engagement, advertising and incentives, pre-existing immunity, and clinical, immunological, and microbiological endpoints. The fourth CHIM meeting focused on CHIM studies being conducted in endemic countries. Over the last ten years we have seen a vast expansion of the number of countries in Africa performing CHIM studies, as well as a growing number of different challenge organisms being used. Community and public engagement with assiduous ethical and regulatory oversight has been central to successful introductions and should be continued, in more communityled or community-driven models. Valuable initiatives for regulation of CHIMs have been undertaken but further capacity building remains essential.

## Abbreviations

AEs: adverse events
AMA: African Medicines Agency
AVAREF: African Vaccine Regulatory Forum
BMGF: Bill & Melinda Gates Foundation
CHIMs: Controlled Human Infection Models
CONEP: Comissão Nacional de Ética em Pesquisa (National Research Ethics Committee Brazil)
CPE: Community and Public Engagement
ECs: Ethics Committees
GMP: Good Manufacturing Practice
IABS: International Alliance for Biological Standardization
ICH-GCP: Good Clinical Practice Guideline of the International Conference on Harmonization
JoSER: National Guidelines for Joint Scientific and Ethical Review
MARVELS: Malawi Accelerated Research in Vaccines, Experimental and Laboratory Systems
MIST: Malaria Infection Studies in Thailand
NDA: National Drug Authority
NRAs: National Regulatory Authorities
PLWH: People living with HIV
PPB: Pharmacy and Poisons Board
SOPs: Standard Operating Procedures
UNCST: Uganda National Council for Science & Technology

## 1 Introduction

Earlier meetings laid the foundations for Controlled Human Infection Models (CHIMs), also known as human challenge studies and human infection studies, including Good Manufacturing Practice (GMP) production of the challenge agent, CHIM ethics, environmental safety in CHIM, recruitment, engagement, advertising and incentives, pre-existing immunity, and clinical, immunological and microbiological endpoints [1–4]. The fourth CHIM meeting was organised in Mombasa, Kenya, from 22 to 23 May 2023 and focused on CHIM studies being conducted in endemic countries. The meeting was hosted by the KEMRI-Wellcome Trust Research Programme where CHIM studies on malaria and *Shigella* have been conducted and are being planned. Many institutions across Africa have conducted or are planning CHIM studies, especially malaria CHIM, and the work is inherently multi-disciplinary.

Wolfram Metzger (Faculty of Medicine, Eberhard Karls University Tübingen, Germany) provided an overview of the historical and ethical aspects of CHIM; especially as scientific ambition can clash with ethics. Experimental infections in humans were often unethical, with subjects not consenting to being challenged and the number of incidents led to stricter regulatory rules [5]. Eventually, this culminated in the 1964 Declaration of Helsinki, compiling a set of ethical principles regarding human experimentation developed for the medical community by the World Medical Association. Similarly, the Good Clinical Practice Guideline of the International Conference on Harmonization (ICH-GCP) is an international ethical, scientific, and quality standard, the purpose of which is to harmonise technical procedures and standards, to improve quality, and reduce the time for a drug to get to the market. This standard emphasises protecting the rights, safety, and welfare of human subjects who participate in studies.

Ally Olotu (Ifakara Health Institute, Tanzania) presented the history of science in Africa. Traditional medicine was a dominant medical system widely accessible, based on beliefs and experiences relevant to specific cultures. This involved the use of plants with medicinal effects.

Evidence is available that African societies were advanced in metallurgy and tool making and were making advances in science and technology. Some examples are the advanced medical and surgical procedures developed in Egypt, and Sankoré University in Timbuktu, where training in basic science, mathematics, physics, chemistry, and business code and ethics was provided. Colonialism disrupted indigenous knowledge systems, and the exploitation of natural resources led to urbanisation, thus exacerbating poor health. The post-colonial era shows a revitalisation of African scientific research, with an emphasis on applied research. However, limited infrastructure and funding, leading to a brain drain, remained a challenge for African research. In the early 1990s, the adoption of ICH GCP guidelines, which established global standards for conducting clinical trials, sparked a surge in research activities in Africa. This increase was fuelled by a rise in international funding and partnerships. Consequently, there was a notable improvement in collaborations between institutions in northern and southern regions, leading to enhanced biomedical facilities and greater access to resources and knowledge exchange.

However, there remains inequity in the distribution of benefits, funding, and career development for African scientists. As an example, 17.5% of the global population lives in Africa but only 2.5% of all clinical trials are conducted in Africa. This is due to a scarcity of well-trained and skilled researchers, a lack of data systems and secure storage infrastructure, and a lack of funding, among other things. This impacts data collection, analysis, and monitoring processes with the perception that there is a reduction in the research outcomes’ overall quality and reliability.

African-led scientific initiatives, like AAS (African Academy of Sciences) and SFA (Science for Africa), are collaborating with global partners to strengthen local skills and capabilities. Their aim is to align research efforts with crucial scientific and health challenges specific to the region, while also promoting local ownership of research activities. This approach seeks to enhance and provide confidence in the overall research output and impact within Africa.

Furthermore, there are regulatory complexities, such as local differences in regulatory requirements, review expertise and pathways, and lack of capacity for new emerging research areas. This leads to delays in the approval process with huge financial and timeline implications. The challenges are being addressed through the strengthening of regulatory frameworks and collaborations, including regional regulatory collaboration (e.g., East Africa), and pan-African regulatory initiatives (African Vaccine Regulatory Forum, AVAREF, African Medicines Agency, AMA). One success story is the malaria RTS,S vaccine trial, where large phase 3 trials were performed in Burkina Faso, Gabon, Ghana, Kenya, Malawi, Mozambique and the United Republic of Tanzania. This African-led initiative supported the development of sites and the training of personnel. Another example is the South-South collaboration between Tanzania and Equatorial Guinea, which managed to accomplish the first-ever clinical trials in a central African country including CHIMs in partnership with northern partners and private sector in Equatorial Guinea and built a mentorship program for Ethics Committee members and local researchers, established a regulatory and ethics review system to support clinical trials, and led to capacity building to support future trials.

## 2 Different challenge agents used in CHIMs in endemic settings

### 2.1 Malaria

Nicholas Day (Mahidol Oxford Tropical Medicine Research Unit (MORU), Thailand) talked about the Malaria Infection Studies in Thailand (MIST). *P. vivax* is less common than P. *falciparum*, but it is more difficult to treat. A standardised inoculum for *P. vivax* is difficult, as it cannot be cultured *in vitro*. MIST1, this first phase of the program of work was to prepare an inoculum under near-GMP from volunteers; *P. vivax*-infected mosquitoes were used to infect naïve volunteers (i.e., intradermal infection). Two banks of vivax challenge strains were produced and MIST2, a dose-finding study, showed that both challenge strains banked had a high attack rate in human volunteers. No serious adverse events (AEs) were observed, although some high-grade AEs were seen. MIST3 will investigate the efficacy of a subunit vaccine (Institut Pasteur) [6]. Initially, significant effort was needed to align regulators, Institutional review boards (IRBs) and community leaders around ethics, but eventually, there were no regulatory or ethical hurdles for approval of MIST.

#### 2.1.1 Discussion of a malaria protocol

##### What is the acceptable limit of risks to volunteers?

For participants there are physical and mental health risks, due to isolation for 46 days. This can be partly mitigated by the possibility of visits by family members. Furthermore, isolation does not take place in hospitals but in houses in low endemic regions, where it is possible to socialise (outdoors) during the day. At night, when the risk of mosquito bites increases, participants must stay indoors.

##### How to mitigate risks for third parties?

The study will be performed in a low transmission area. When it is necessary for a participant to leave before the end of the study, treatment is completed before withdrawal.

##### What are the risks associated with treatment?

Sub-curative treatment is provided in the middle of the study to reduce morbidity but still allow transmission. Complete treatment is provided at the end of the study. This must be communicated clearly, so the participants understand. Nevertheless, an infection in a CHIM study would pose a smaller risk than a natural infection due to the controlled circumstances and close observation.

##### How is post-trial follow-up done?

This is six months after discharge. How can AEs be documented? Do participants have access to care? Generally, participants have better access than the general population, which is an advantage of participation. Follow-up after protocol (beyond the study period) − IRB can ask for follow-up phone calls up to five years after completing the study. The preferred follow-up is passive, with the initiative coming from the participant. Alternatively, this can take place through medical databases.

##### How to ensure that volunteers understand the risks?

Communication needs to be tailored, to communicate at the right educational level. This can lead to a minimum educational level for the participants, to make sure they understand the risks.

### 2.2 Shigella

Kawsar Talaat (Johns Hopkins Bloomberg School of Public Health, U. S.A.) discussed the *Shigella* CHIM. *Shigella* was the second leading cause of mortality in 2016 among all ages [7]. The high global burden and rise in AMR has led to *Shigella* being a PDVAC priority pathogen and identified as a priority for needing a vaccine. A *Shigella* CHIM in Kenya can help to accelerate vaccine development by allowing those vaccines and monoclonal antibodies currently in development to be tested for efficacy. To develop a model, careful evaluation in healthy adult human volunteers is essential to determine the dose, the optimal fasting/buffer regimen [8], the attack rates (and their reproducibility) [9], the time to onset of symptoms or disease, and development of harmonised clinical endpoints for use in subsequent studies [10].

#### 2.2.1 Discussion of a Shigella protocol

##### What is the acceptable limit of risks to volunteers?

Differences exist between pathogens and study design, ranging from pre-symptomatic endpoints to disease endpoints. *Shigella* challenge is strictly a disease model.

##### How to mitigate risks for third parties?

This also encompasses the environment and study personnel. Risk education is necessary in the community, especially in multigenerational homes (as infants and elderly people are more susceptible). However, these studies have to be done in an inpatient facility as the volunteers are too sick to stay at home. They are then treated before they go home to minimise any risk to their household members.

##### How will the reporting and management of adverse events (AEs) be done?

The level of reporting of AEs should be in line with the country’s regulations. A passive approach is not very systematic. As a standard, AEs are collected actively by the study team; volunteers who get sick receive medical care from the study team.

##### How to ensure that volunteers understand the risks?

Explain and ask to speak back, as a way to gauge understanding of the study, including its risks and benefits. This enhances equity in participation, as it does not depend on literacy.

### 2.3 Streptococcus pneumoniae

Kondwani Jambo (Malawi-Liverpool-Wellcome Trust Clinical Research Programme, Malawi) talked about the *Streptococcus pneumoniae* (Spn) carriage model. The model was set up in Liverpool and then transferred to Malawi (Malawi Accelerated Research in Vaccines, Experimental and Laboratory Systems, MARVELS). Here, high carriage and invasive disease are still observed, despite introduction of PCV13. The CHIM is being developed for Spn serotype 3, which is increasing worldwide, although it is incorporated in PCV13. Further interest is in setting up CHIM in a high-risk group: e.g., people living with HIV (PLWH), as their immune status will affect the response to the vaccine. Carriage is higher in PLWH, increasing the likelihood of shedding/transmitting Spn.

#### 2.3.1 Discussion of a Streptococcus protocol

##### What is the acceptable limit of risks to volunteers?

As treatment is available, the risks to the individuals are limited. As the volunteers are not screened after treatment, there is a limited risk for third parties, especially in vulnerable populations, such as PLWH and elderly people. Volunteers should limit interaction with members from these groups. A test of clearance of pathogens would be preferred to avoid carriage. Antibiotic treatment may drive antimicrobial resistance (AMR) and should therefore be avoided. Colonisation with e.g., Spn 6B, may be beneficial, and rescue treatment with antibiotics may put individuals at risk of harm. Volunteers are given a choice of whether or not to be treated.

##### How to ensure that volunteers understand the risks?

Most volunteers are students, with a medical background, so there is a lower risk for non-understanding.

##### How is post-trial follow-up done?

Volunteers actually prefer to stay engaged, so this is not an issue.

### 2.4 Schistosoma mansoni

Moses Egesa (MRC/UVRI and LSHTM Uganda Research Unit, Uganda) discussed the schistosomiasis CHIM that has been introduced in Uganda. More than 90% of all *Schistosoma* infections take place in sub-Saharan Africa. Although treatment is available, re-infection occurs often and rapidly. Hence, a vaccine would be valuable. The vaccine candidate Sh28GST failed in trials, leaving three others (Sm14, SmTSP2, and Smp80) in the pipeline. A CHIM for Schistosomiasis in Uganda can help in selecting the best vaccine for this population to move forward to Phase III clinical trials reducing risk of failure at this stage. Conducting these studies in endemic settings where pre-immunity and other comorbidities such as malaria and HIV exist, gives confidence to developers should efficacy be shown. The schistosomiasis model has been established in the Netherlands with an initial safety and dose-finding study performed, challenging participants with 10, 20, or 30 cercaria. To transfer the model from the Netherlands to Uganda, production of the challenge materials (that involves infecting a single snail with a single miracidium) in Uganda is necessary [11]. A local snail species from Lake Victoria was used however preliminary work indicates that this species is resisting infection. Different snails will be investigated from other sources e.g., another local species will be tested from Uganda’s second biggest freshwater lake, Lake Albert. Also, the possibility of importing the snail successfully used in the Dutch CHIM is being considered, although this solution comes with its own set of challenges.

#### 2.4.1 Discussion of a schistosomiasis CHIM protocol

##### What is the acceptable limit of risks to volunteers?

Risk for participants is limited due to use of single-sex cercariae, and risk of spread to the community is limited. Treatment failure with praziquantel can happen. In that case, a second dose will be needed or other options of treatment such as antimalarials (artesunate/lumefantrine) or pre-sumptive treatment.

##### How can loss-to-follow-up be minimised?

In an outpatient model, this risk is bigger. Providing facilitation may pre-empt this but raises ethical dilemma regarding undue influence.

##### How will the reporting and management of AEs be done?

Soliciting and reporting AEs will be according to timelines as described in the protocol [11]. Post-trial follow-up is not deemed necessary.

##### How to ensure that volunteers understand the risks?

Understanding of risks by volunteers who may be of low scientific literacy would require translating the information to local language and a test of comprehension of the protocol checklist to verify understanding. Given that it is a complex model even for experts, how we inform non-experts is very critical to the implementation of CHIM for schistosomiasis.

##### Why is a CHIM useful in the development of interventions?

A CHIM can be used to test drugs against schistosomiasis including repurposing anti-malarial drugs. CHIM is useful for developing diagnostics as it may simulate a situation of low infection intensity in a community. Finally, it is possible to perform exploratory immunological research on correlates of protection.

##### What potential issues/challenges need to be addressed?

Equity issues in remuneration arise when doing studies in high-income countries (HIC) and low- and middle-income countries (LMIC). One area of debate is how to define a fair compensation. Including locals to gauge expectations is an option to mitigate the issue.

### 2.5 SARS-CoV-2

Chris Chiu (Imperial College London, UK) talked about setting up the first COVID-19 CHIM (COVHIC001) during the early pandemic. The initial rationale was to develop the CHIM to quickly select the most promising vaccine candidates if first-generation vaccines were either ineffective or conferred only modest protection. However, by the time that the study was ready for ethical review, a number of vaccines had already proved in phase 3 trials to be highly effective against hospitalisation and death. Nevertheless, it was argued that the CHIM would still be useful in understanding aspects of the disease and immunity that could not be obtained from field studies. Furthermore, as it became apparent that approved vaccines had limited efficacy in preventing transmission and relatively short-lived protection, the role of the CHIM has re-emerged as a way to provide efficacy data of novel vaccine candidates when phase 3 studies are no longer feasible due to low attack rates in the immune population.

In the first study, risks were reduced by only including participants between 18 and 30-years of age and doing thorough pre-challenge clinical assessments. Participants were quarantined, with close clinical monitoring and long-term follow-up. The results of the first study showed that the incubation time was only two days, much shorter than previously estimated. Moreover, extremely high peak viral loads were rapidly reached in all infected individuals, irrespective of symptoms, which were mostly mild-to-moderate in nature. Decreased sense of smell was common and mostly short-lived, although in a few participants it continued well beyond other symptoms before fully resolving. Viral emission was tested in breath, air, and on surfaces, showing a large diversity between participants, from individuals potentially behaving like “super-spreaders” to those who emitted no virus in the environment despite having high levels in the nose and throat. The strongest correlation was observed with viral load in the nose (as opposed to the throat). The findings suggested that transmission could be effectively interrupted if lateral flow antigen tests were systematically implemented and acted upon.

#### 2.5.1 Discussion of a Sars-CoV-2 protocol

##### How will the post-trial follow-up of participants be done?

Because of the uncertainty around long-term sequelae, e.g., long-COVID, long-term follow-up is needed − at least 180 days. Similarly, pregnancies occurring during or shortly after the study should be observed until after delivery.

##### Could this have been done in an LMIC setting?

What extra challenges would have existed? Would family consent beyond participant consent be needed, especially if a disease is new? Should such a CHIM be prioritised in an LMIC setting with a weak health setting? The CHIM participants may take up space (i.e., ICU beds) that is needed for actual patients. This needs to be thought through before the situation arises. Engagement is crucial, otherwise, it will result in distrust if interventions are forced on people. Special insurance coverage is needed because the long-term effects are largely unknown.

### 2.6 Zika virus

Anna Durbin (Johns Hopkins Bloomberg School of Public Health, U. S.A.) discussed the Zika CHIM, which could contribute to vaccine and drug development, but also provide a better understanding Zika infection in humans and Zika transmission. Initially, the CHIM was rejected when the epidemic was in full swing. But with Zika waning, the CHIM was approved in October 2019. As the most serious consequence of Zika infection is microcephaly, the risks were limited by only including non-pregnant women below 40 years of age, without history of neurological diseases, in an inpatient setting. They were tested for pregnancy before and at the start of the study and were required to use highly effective birth control and barrier contraception for two months after the study. Endpoints of the dose-finding study were at least 80% infection after challenge and viral infection on at least two (consecutive) days, preferably with mild symptoms (rash). Other symptoms were nonpurulent conjunctivitis, arthralgia, and retro-orbital pain. Volunteer have been enrolled into two cohorts (seven in each of the two viruses tested). Of the first five volunteers who received ZIKV-SJRP, the lowest dose (100 pfu) 100% got infected, with rash as a good indicator. The remaining seven volunteers will be enrolled into each cohort (five will receive ZIKV-SJRP, five will receive ZIKV-Nicaragua, and four will be in the placebo arm). Dose escalation will probably not be needed but will need to be discussed with the Data Safety Monitoring Board.

#### 2.6.1 Discussion of a Zika virus protocol

##### What is the acceptable limit of risks to volunteers?

Although still theoretical rather than experimental, a Zika infection may elicit antibodies that are cross-reactive with dengue and increase the risk of antibody-enhanced disease, when infected with dengue afterwards. The Zika CHIM also has a risk of spreading the virus to third parties through sexual contact or via mosquitoes. Therefore, the CHIM should first be used in non-endemic countries. But it could also be done in regions where partial immunity has been acquired after the epidemic. Furthermore, there is a risk due to limited knowledge of viral reservoirs. Finally, it is a risk and challenge to communicate why only women are enrolled in the study, depending on the level of autonomy of the woman in deciding to participate. Therefore, the cultural context should be taken seriously.

Community and public engagement (CPE) is essential at an early stage, especially as Zika is not well known in many populations.

##### How cured should the participant be to be let go?

To be released, a participant should have no symptoms, a significant viral load reduction, and preferably no virus left, depending on the diagnostic test used. Two days of sequential negatives may be needed for release to provide more reassurance.

##### How will the reporting and management of AEs be done?

Neuropathic diseases, sexual transmission, and immune complications can occur. Follow-up through active surveillance should be a minimum of 28 days.

##### How will the post-trial follow-up of participants be done?

Good communication of signs and symptoms to participants (and their families) is necessary, to make sure they will contact the study team when symptoms occur.

##### What potential issues/challenges would need to be addressed?

A CHIM can lead to reputational risk − having an unrelated outbreak while doing a CHIM will harm the research and the confidence in science in general.

### 2.7 Dengue virus

Do Van Dung (University of Medicine and Pharmacy, Ho Chi Minh City, Vietnam) addressed the Vietnamese students’ perception of clinical research, including CHIMs. In Vietnam, deliberate infection is illegal, so CHIM studies cannot currently be performed. Besides, other measures than vaccines (as a result of CHIM studies), e.g., personal hygiene for Shigella, may be equally effective and more equitable. However, for dengue, this is not a solution, and the global burden is high. However, a vaccine for dengue is also challenging due to antibody-enhanced disease. A CHIM may help in developing and testing an appropriate vaccine and in treating dengue, as appropriate drugs are currently lacking. Besides the legal issue, the researchers aimed to understand the readiness of the population for CHIM studies, and the barriers that may still exist. Four key actions were undertaken, a science café to provide information on CHIMs, a science debate where CHIMs were discussed by students playing different roles (e.g., parent, researcher). The knowledge acquired was used to prepare a questionnaire to investigate their social environment, as participatory action research. Finally, the data collected was presented as a poster, video, or website, for a competition.

#### 2.7.1 Discussion of a dengue protocol

##### What is the acceptable limit of risks to volunteers?

There is an increased risk of antibody-enhanced disease in endemic versus non-endemic regions. This leads to the importance of rigorous screening before participation. However, it may also be a potential risk to frequent travellers living in non-endemic areas. Therefore, it would be good to have a test for previous dengue exposure. Some symptoms can remain for a long period, beyond the end of the trial. Participants need to be well informed about this.

##### How will the reporting and management of AEs be done?

SAEs need to be reported in accordance with national guidelines. Online diaries may be useful for reporting AEs, although this may be a burden on the researchers. Long-term sequelae may be detrimental to the view on CHIMs in the population.

##### How will the post-trial follow-up of participants be done?

Long-term follow-up is necessary for safety and surveillance.

##### How to ensure that volunteers understand the risks?

Audio-visual materials may help for increased understanding, a comprehension test of understanding afterwards is needed to make sure that participants understand.

##### Why is a CHIM useful in the development of interventions?

Correlates of protection are an important reason to perform CHIM, as is the up- or down-selection of vaccines.

### 2.8 Discussion

During the discussion of the models, further details about the manufacture of the different challenge agents were requested. Overall, there was a consensus that the use of a local strain is an advantage. For *P. vivax*, no standard inoculum is available as the organism is difficult to culture. For *Shigella*, GMP manufacture of the challenge strain is used, with minimal handling, which increases consistency. For Spn, each dose is checked to ensure it contains the right challenge strain. For *Schisto-soma*, the Leiden challenge strain is currently used, and an Ugandan strain is under development.

A second question was about selection of participants and how informed consent was obtained. For *P. vivax*, online adverts are used. Interested people (undergraduates and graduates) are informed in groups. Pros and cons are discussed, followed by a test of understanding. When this test is passed, informed consent is signed. For *Shigella*, adverts are used as well. Three face-to-face meetings take place before enrolment. Risks are discussed, including the lack of personal benefits for the volunteer. No minimum level of education is required. For Spn, 2-h information sessions are organised. If interested, volunteers are invited to a face-to-face meeting during which the information sheet is discussed which then leads to a personal appointment, to discuss risks and the limited right of withdrawal. Word of mouth is helpful, with an increasing number of volunteers seen over time.

The third question moved onto conducting these studies in endemic populations and a query on how immune do we want our study population to be? The study population should be representative of the target population. As a vaccine will generally be given before infection, it is important to compare naïve with non-naïve populations.

The different models show many commonalities but also pathogen-specific issues.

Funding cannot be partial; a study needs to be fully funded to ensure follow-up afterwards.

Test of understanding − if failed, can it be done again? The participants need to understand, if not, they cannot be enrolled. It is necessary to be sure that volunteers know what they are doing/signing up for. A questionnaire may be selective and inequitable. Inform and hear back is better, as it does not rely on literacy, although it is difficult to grade systematically. The community also needs to be informed. A participant may consent, but the community may object. Community engagement provides a longer time period to fully understand the study.

Communication of the study results must extend not only to other researchers but also to the participants and broader communities, so they understand the benefits. This also helps to convince communities of future research or the need for high vaccine uptake.

The discussion also included the consideration of shedding of infectious versus non-infectious particles. For SAR-CoV-2, the culture assay, which detects infectious particles, shows 10^e5^ times lower viral load and shorter duration of infection than PCR, which detects both infectious and non-infectious material. However, the culture assay has limitations to detect infectious virus and a negative test may not be a good indicator of lack of infectiousness. In contrast, ZIKV can also be cultured, although this is a more time-consuming assay than PCR. Men were shown to shed viral material in semen detected by PCR. But they did not infect their partners, showing that it was not live, infectious virus. Clearly, culture is more useful here.

Looking at mucosal immunity in people with low and high SARS-CoV-2 viral loads, it is evident that T-cells in the blood and IgA and IgM in the mucosa are related to a rapid decline in viral loads. A transcriptomic approach, not necessarily hypothesis-driven, is used to also look at innate immunity. However, the methods need to be improved, and sampling of the upper respiratory tract must be done more consistently.

In outpatient studies, what is the monitoring plan for the people in the household of the participant? Similarly, for vector-borne diseases, are there procedures for handling the vectors to make sure they do not escape into the environment? These are issues that need further discussion.

## 3 Requirements to perform CHIM studies in endemic settings

Primus Chi and Dorcas Kamuya (KEMRI-Wellcome, Kenya) discussed the ethical aspects of CHIM studies in Africa. CHIM studies raise important ethical issues, especially in LMIC settings such social acceptability of deliberate infection; ensuring valid informed consent (which should be based on an appropriate understanding of the study); inadequate guidance on appropriate compensation levels for in-conveniences experienced in being part of these studies; limited right of withdrawal from the study, due to the risk of transmission; and managing the risks of third-party infections. Nevertheless, CHIM studies can be performed; in Kenya, two studies have been completed, while four studies are ongoing or planned. Social science research has been conducted to prepare the ground for these studies, leading to important insights that have contributed to policy and practice. Major themes that have emerged from the research include study payments, benefits and burdens, and women’s participation. This has contributed to the design and conduct of CHIM studies in Kenya, including the introduction of demonstration tools for the specific volume of blood drawn; staggering of compensation for participation (from end-of-study lump sum to weekly payments) in CHIM studies; inclusion of participants from populations where high literacy is not an a priori criterion, leading to better inclusivity in research participation; and ongoing inclusion of uncovered benefits and burdens as part of the informed consent process.

A scoping review of social science research and engagement studies around CHIM studies showed that the conditions that have to be met to increase social acceptability of CHIM studies are participants’ safety, potential to benefit study population, voluntary informed consent, fair participant selection, adequate medical care, adequate community engagement, fair compensation and reimbursement, and a strong regulatory system. Other major issues that affected social acceptability included potential reactions from study communities and risks of third-party infection.

Factors influencing participation in CHIM studies were monetary compensation and reimbursement, access to a ‘free’ health check, and altruism, whereas factors influencing decision-making included study-associated incentives, trust in the research institution and review system, perceived associated burdens, and understanding of the study procedures and the consenting process.

Engagement with key stakeholders and the public is critical in the needs assessment, development, design and conduct of CHIM studies. The right stakeholders should be engaged long before study commencement. This engagement must be carefully designed to target specific stakeholder groups, and address concerns raised by the stake-holders. Afterwards, debriefing sessions provide opportunities for improvements of future engagement activities.

Overall, although CHIM studies raise important social and ethical issues, they can be undertaken in Africa with the specific issues addressed.

Melissa Kapulu, (KEMRI-Wellcome Trust Research Programme, Kenya), discussed the use of CHIM in endemic populations with studies undertaken in Kenya as a case study for these models. Until recently, most of the CHIM work in Africa was limited to six countries and focused on malaria [12]. Now, more models are being introduced and more sites in more countries are working on CHIMs. Community and Public Engagement (CPE) is integral to the successful conduct of CHIM studies. This includes engaging with a variety of identified stakeholders including and not limited to secondary and university students, community representatives, mainstream media, and social media through influencers.

One advantage of doing studies in Kenya is that it allows the inclusion of healthy semi-immune adults with varying degrees of immunity [13]. This has shown that past exposure to malaria, as evidenced by location of residence, in some Kenyan adults can completely suppress *in vivo* growth of a parasite strain [14,15]. The malaria CHIM was used to look at vaccine efficacy in a low-exposure population and quickly demonstrated the down-selection and importance of the route of administration in determining efficacy of a candidate vaccine.

The second model which is currently being introduced at KEMRI-Wellcome is the *Shigella sonnei* model. Ethics, regulatory, and CPE preparations started early to allow to for the implementation of dose-finding studies to induce disease safely and reproducibly in ≥60% of healthy adult Kilifi volunteers. To account for pre-existing immunity, a seroprevalence study is being undertaken in three different settings to inform level of pre-existing immunity considerations for enrolment into the study. Once the model is successfully implemented, a double-blind, placebo-controlled vaccine efficacy study is planned.

Noni Mumba (KEMRI-Wellcome, Kenya) discussed CPE in challenge trials. Community Public Engagement is necessary at an early stage, to make sure that feedback can be incorporated in the study protocol. But after approval further communication remains necessary. This includes education of the study staff, as they will be asked questions throughout the study and must be ready to provide answers. Targets for communication are local communities (including a community advisory board), and policy makers. Real-life stories of participants are valuable to inform and interest potential new participants.

Roli Mathur (Indian Council of Medical Research, Bioethics Unit, India) discussed proposed national ethical guidelines on conduct of controlled human infection studies in India which are being drafted by ICMR. She indicated that guidelines are needed to support researchers as well as the ethics committee to create a standard of research and a framework for the protection of participants as India plans to begin CHIM studies. Guidelines should identify ways to provide the benefits, mitigation of risks and ways to deal with the specific challenges related to CHIMs. To perform a CHIM, expertise in infectious diseases, immunology, epidemiology, and pharmacology is needed, preferably combined with expertise in conducting clinical trials. Volunteers should be healthy young adults, belonging to middle to higher socioeconomic strata. They must at least be graduates/well educated to have good understanding about the research requirements. Details of any payments to be made should preferably be disclosed only after they agree to participate in order to avoid any undue inducement related to monitory gains related to participation in research. The reason for participation should be altruism alone and there should be no coercion involved. The research team as well as ethics committee members require appropriate training in order to have better understanding of the issues involved. Informed consent requires due care and may be followed up with a test of understanding. No CHIM studies can be performed on any vulnerable persons or groups, such as children, pregnant women, or people with comorbidities. Community engagement and public assurance is key to deal with fear and mistrust in the community. As India is a major drug and vaccine manufacturer, with local endemic diseases, the advantages of being able to perform CHIM studies in India are clear, but under tight regulation. The protocol must be thoroughly reviewed and approved and to have built in provisions for robust monitoring/oversight, including a DSMB and institutional biosafety committee. Guidelines to perform CHIM studies are under development by a multidisciplinary expert group.

## 4 Regulation of CHIMs in endemic settings

Frank Sinyiza (National Health Sciences Research Committee, Malawi) explained the regulatory system in Malawi. The National Health Sciences Research Committee reviews all health research proposals, especially those of national interest. These include vaccine trials, stem cell research, cloning research and CHIMs. CHIMs are scrutinised for the local capacity to perform them appropriately, including personnel, infrastructure, diagnostic capacity, and AE monitoring. Furthermore, the potential risk of exploitation of participants, safety issues (for participants, staff, and third parties), and insurance and indemnity cover are reviewed. Finally, reimbursement and compensation, as well as community perception are discussed. So far, several CHIMs have been approved, which are now in various stages of development. Recommendations toward the future are to strengthen local capacity in Malawi both in study conduct, but also in regulation of CHIMs, to enhance community engagement, and to develop an ethical framework for CHIMs. Finally, countries that participate in CHIMs should be prioritised and supported to roll out the vaccines or drugs that are investigated in those CHIMs.

Mikal Ayiro (Pharmacy and Poisons Board, Kenya) shared the Pharmacy and Poisons Board (PPB) experience with CHIMs from Kenya. All clinical trials, including CHIMs will pass the PPB for approval to ensure the quality, safety, efficacy, and the accuracy of information. When reviewing proposals, the PPB will look at regulatory and other considerations that include ethical, technical, and governance. The PPB is often seen as a “gatekeeper” and only involved during submissions for approval. It is of great importance to engage the PPB early enough i.e., at the start, throughout the development process and after finishing the CHIM development and leverage on their expertise during the different stages/phases. Innovation comes from the scientists and other stake-holders who also must drive the engagement with the PPB. Mikal Ayiro encouraged having the PPB-regulator as a partner and collaborator, who provides regulatory strategy, knowledge, expertise, and intelligence to the CHIM development team, preparing the regulatory aspects with the scientists and other stakeholders.

Winfred Nazziwa (Uganda National Council for Science & Technology (UNCST), Uganda) shared the regulatory experience on CHIM from Uganda. The number of research studies registered and approved at UNCST has grown significantly over the last decades. Each study must be reviewed and approved by one of the 34 accredited RECs in the country and UNCST. Clinical trials are additionally reviewed by the National Drug Authority (NDA). The discussion about CHIMs started in 2017, followed by capacity strengthening initiatives, including a visit to Leiden, where the CHIM for *Schistosoma* was developed. In 2019, the first CHIM application was submitted for review to the research regulatory bodies in the country. The CHIM application was reviewed in a joint review mechanism, using the AVAREF joint review guidelines. In November 2020, the schistosomiasis CHIM was approved by UNCST. Based on the AVAREF joint review guidelines, UNCST has developed National Guidelines for Joint Scientific and Ethical Review (JoSER) of research. In this process, the research application is reviewed jointly by the NRAs (UNCST, UNHRO and NDA), RECs, subject matter experts and appropriate stakeholders in a given field. The joint review mechanism aims to optimise review timelines, enhance the quality of the review, exchange and validate findings about the application, and act as a capacity strengthening platform. Challenge agents are not considered as medicines or biologics, therefore are not regulated in the same way as medicinal products. A CHIM study only requires drug authority approval if it is a clinical trial of a drug or drug-related product. The responsibility for ensuring that the CHIM study is conducted suitably, and the challenge agent is manufactured and used appropriately lies entirely with the UNCST, REC, and the National Biosafety Committee.

As CHIMs are still comparatively new, the following actions are recommended: continuous strategic effective community engagement, capacity strengthening activities for the key stakeholders, development of clear guidelines/frameworks for review of CHIMs, harmonised guidelines/principles for designing, developing, and manufacturing challenge agents to align with other forms of clinical research. Consultation with other regulatory bodies with experience in overseeing CHIMs should be encouraged.

Jacqueline Rodgers (Secretariat for African Vaccine Regulatory Forum, AVAREF) explained the AVAREF Scientific Advice Procedure and AVAREF Joint Review Procedure for Multi-country Clinical Trials. AVAREF is a network of National Regulatory Authorities (NRAs) and Ethics Committees (ECs), which was established in 2006 by WHO to build capacity and improve the harmonization of practices in support of product development. Its mission is to help NRAs, ECs, and sponsors to achieve consensus on regulatory and ethics questions surrounding research and development of medical products. The primary aim is to ensure timely regulatory evaluations and decision-making processes on clinical trial applications. To date, the AVAREF network connects regulators and ECs from 55 member states in Africa. Three types of AVAREF joint review are possible, regular with an expected timeline of 60 working days, expedited (30 working days), and emergency (10–15 working days). This is possible through early scheduling of meetings, expedited response of all stakeholders, efficient communication through digital tools, strengthened collaboration, and adaptation of the process, including electronic submission and completion of administrative processes.

## 5 CHIM introductions

Rodrigo Correa-Oliveira (Fiocruz, Brazil) talked about the regulatory and ethical hurdles of implementation of hookworm challenge trials in Brazil. The intention is to transfer the hookworm CHIM developed at the George Washington University (USA) to Brazil. The project entails three parts, 1) the transfer of the CHIM by establishing a donor cohort, 2) a dose escalation study, and 3) a vaccine trial-challenge study for accelerated efficacy testing. To prepare for these studies staff were trained in Good Laboratory Practice, the laboratory was renovated for GMP-like hookworm larvae production, carefully documenting every step of the process, SOPs for larvae production were prepared, clinical protocols were transferred and adapted, and clinics were prepared in low- and high-endemic areas. Finally, ethical and regulatory packages were prepared and submitted to a local EC. The members, however, did not feel qualified to review and deferred the review to CONEP, the national authority. After several meetings and rounds of review the project was still not approved, despite support from the Ministry of Health and other government organisations. CONEP did not consider the study to be unethical, but the main concern was related to performing the CHIM in populations living in endemic areas. It was felt that these studies could be conducted in individuals with higher levels of education, who had been previously infected or were currently infected. Recently, the study has received co-funding from the Brazilian Government which permits a new ethical review submission via the EC of the Federal University of Minas Gerais (Belo Horizonte). This new EC is waiting for certification by CONEP, after which the proposal can be submitted, toward the end of 2023.

Elizabeth Chandler Church (Fred Hutchison Cancer Center, Seattle, U.S.A.) explained the need for a tuberculosis (TB) challenge model. TB is an important cause of infectious disease related mortality worldwide. The existing vaccine, BCG, prevents disease in children but minimally impacts transmission and can cause infection in immunocompromised individuals. Difficulties with the diagnosis of TB make phase II and III trials for vaccines very expensive (due to the large sample size required). Finally, drug development for TB has been slow due to high associated costs. It is ethically difficult to use *Mycobacterium tuberculosis* in a challenge model, due to challenges in diagnosis, duration of treatment, and possibility of transmission. On the other hand, BCG is a live attenuated version of *M. bovis*, and has already been approved as a childhood vaccine. It can therefore be used as a challenge organism, as has been shown previously [16–18]. However, challenges in translation to *M. tuberculosis* remain; correlates of immunity are not fully understood, infection does not always lead to disease, and is difficult to measure. From an experimental point of view, measuring bacterial burden and immune response is difficult because culture is slow, prone contamination and overgrowth by skin bacteria, and isolation is variable, based on location of the biopsy relative to the BCG lesion. Molecular viability testing (MVT, a method of assessing the viability of bacterial cells by exploiting the ribosomal RNA synthesis pathway) may shorten time to diagnosis compared to culture, and hence, accelerate treatment. An initial study comparing culture with MVT showed similar patterns for colony-forming units (CFU)/biopsy and MVT peak ratio, indicating a correlation between CFU and MVT. Treatment with isoniazid showed no effect on bacterial load but did show an increased number of B-cells and CD14-positive cells (innate immune cells). Next steps include investigation of correlations between biopsy and systemic responses, evaluation of MVT using rifampin as treatment, non-invasive measurements of bacterial burden with BCG Glow, and the use of challenge models for host directed therapeutics in TB. BCG is not the perfect model for vaccine and drug development. Regrettably, it is still unclear whether it will be possible to move to *M. tuberculosis* as challenge agent, due to the ethical challenges around diagnosis, treatment, and transmission.

## 6 Guiding principles for funders of CHIM studies

Claudia Emerson (McMaster University, Canada), Shobana Bala-singam (Wellcome Trust, UK), and Gary Means (Bill & Melinda Gates Foundation, US) discussed guiding principles for funders of human challenge studies. Initially CHIMs were mainly conducted in HIC, whereas the biggest need for the fight against infectious diseases is in LMIC. To perform CHIMs in LMIC, regulatory and ethical processes must be established locally. To ensure sustainability, guiding principles are needed, particularly with new funders coming on board and with the increasing number of studies in endemic settings being established. These can express values and commitments, set the tone and norms for conduct of studies, manage expectations of the various stakeholders, shape policy development, e.g., through sharing of SOPs and study data, and strengthen capacity and confidence, which will contribute to trust-building. The five guiding principles are:

- Advance high-quality research that is scientifically and ethically justified
- Ensure the safety of participants and communities
- Engage meaningfully with participants, communities and the public
- Support opportunities to enhance resource capabilities and maximise value of studies
- Share knowledge and data efficiently and responsibly

CPE − should we shift toward community-led and community-driven models of engagement, to make sure that the experience with CHIMs in the community is not lost and used to its fullest? The principles are open to that, it does not have to be driven by the researchers, but they have to be open to the community, open to co-design.

Although CHIMs are among other human studies, including clinical trials, for which guidance exists, having a specific set of guiding principles for CHIMs will add to trust in these studies.

The work on the guiding principles is not done, they can evolve over time, to reflect new insights.

## 7 Conclusion

Ten years after the first CHIM study in Africa, a vast expansion of the number of countries performing CHIM studies, as well as a growing number of different challenge organisms used, can be observed. CPE has been at the root of successful introductions and should be continued, in more community-led or community-driven models. Valuable initiatives for harmonization and regulation of CHIMs both in LMICs and HICs have been undertaken but further work is needed to drive the utility of these studies and acceptance of data by product developers and regulators which will provide the impetus for further investment.

## References

[1] Sheets RL, Fritzell B, Aguado de Ros MT (2016). Human challenge trials in vaccine development: strasbourg, September 29 - October 1, 2014. Biologicals.

[2] Baay MFD, Richie TL, Neels P (2019). Human challenge trials in vaccine development.

[3] Bekeredjian-Ding I, Van Molle W, Baay M, Neels P, Conrad C, van Diepen A (2020). Human challenge trial workshop: focus on quality requirements for challenge agents, Langen, Germany. Biologicals.

[4] Pollard AJ, Sauerwein R, Baay M, Neels P, Balasingam S, Bejon P (2020). Third human challenge trial conference, Oxford, United Kingdom, February 6-7, 2020, a meeting report. Biologicals.

[5] Metzger WG, Ehni HJ, Kremsner PG, Mordmuller BG (2019). Experimental infections in humans - historical and ethical reflections. Trop Med Int Health.

[6] Hou MM, Barrett JR, Themistocleous Y, Rawlinson TA, Diouf A, Martinez FJ (2022). Impact of a blood-stage vaccine on <em>Plasmodium vivax</em> malaria. medRxiv.

[7] Khalil IA, Troeger C, Blacker BF, Rao PC, Brown A, Atherly DE (2018). Morbidity and mortality due to shigella and enterotoxigenic Escherichia coli diarrhoea: the global burden of disease study 1990-2016. Lancet Infect Dis.

[8] Talaat KR, Bourgeois AL, Frenck RW, Chen WH, MacLennan CA, Riddle MS (2019). Consensus report on Shigella controlled human infection model: conduct of studies. Clin Infect Dis : Off Publ Infect Dis Soc Am.

[9] Kaminski RW, Pasetti MF, Aguilar AO, Clarkson KA, Rijpkema S, Bourgeois AL (2019). Consensus report on Shigella controlled human infection model: immunological assays. Clin Infect Dis : Off Publ Infect Dis Soc Am.

[10] MacLennan CA, Riddle MS, Chen WH, Talaat KR, Jain V, Bourgeois AL (2019). Consensus report on Shigella controlled human infection model: clinical endpoints. Clinical infectious diseases : an official publication of the Infectious Diseases Society of America.

[11] Abaasa A, Egesa M, Driciru E, Koopman JPR, Kiyemba R, Sanya RE (2023). Establishing a single-sex controlled human Schistosoma mansoni infection model for Uganda: protocol for safety and dose-finding trial. Immunother Adv.

[12] Kibwana E, Kapulu M, Bejon P (2022). Controlled human malaria infection studies in africa-past, present, and future. Curr Top Microbiol Immunol.

[13] Kapulu MC, Njuguna P, Hamaluba MM (2018). Controlled Human Malaria Infection in Semi-Immune Kenyan Adults (CHMI-SIKA): a study protocol to investigate in vivo Plasmodium falciparum malaria parasite growth in the context of pre-existing immunity. Wellcome Open Res.

[14] Kapulu MC, Njuguna P, Hamaluba M, Kimani D, Ngoi JM, Musembi J (2021). Safety and PCR monitoring in 161 semi-immune Kenyan adults following controlled human malaria infection. JCI Insight.

[15] Kapulu MC, Kimani D, Njuguna P, Hamaluba M, Otieno E, Kimathi R (2022). Controlled human malaria infection (CHMI) outcomes in Kenyan adults is associated with prior history of malaria exposure and anti-schizont antibody response. BMC Infect Dis.

[16] Harris SA, Meyer J, Satti I, Marsay L, Poulton ID, Tanner R (2014). Evaluation of a human BCG challenge model to assess antimycobacterial immunity induced by BCG and a candidate tuberculosis vaccine, MVA85A, alone and in combination. J Infect Dis.

[17] Minassian AM, Ronan EO, Poyntz H, Hill AV, McShane H (2011). Preclinical development of an in vivo BCG challenge model for testing candidate TB vaccine efficacy. PLoS One.

[18] Blazevic A, Xia M, Turan A, Tennant J, Hoft DF (2017). Pilot studies of a human BCG challenge model. Tuberculosis.

